# Succinum extracts inhibit microglial-derived neuroinflammation and depressive-like behaviors

**DOI:** 10.3389/fphar.2022.991243

**Published:** 2022-08-16

**Authors:** Ji-Yun Kang, Dong-Cheol Baek, Chang-Gue Son, Jin-Seok Lee

**Affiliations:** Institute of Bioscience and Integrative Medicine, Dunsan Hospital of Daejeon University, Daejeon, South Korea

**Keywords:** succinum, neuroinflammation, microglia, CX3CR1 chemokine receptor, depressive symptoms, depression

## Abstract

Microglia are emerging as important targets for the treatment of neuropsychiatric disorders. The phagocytic microglial phenotype and the resulting neuroinflammation lead to synaptic loss and neuronal cell death. To explore potential candidates that inhibit microglial hyperactivation, we first investigated ten candidate extracts of traditional Chinese medicine (TCM) using lipopolysaccharide (LPS)-stimulated BV2 microglial cells. Among the candidates, *Pinus spp.* succinum extract (PSE) was superior; thus, we further investigated its pharmacological activity and underlying mechanisms both *in vitro* and *in vivo*. Pretreatment with PSE (10, 20, and 40 μg/ml) attenuated the increases in inflammatory factors (nitric oxide and tumor necrosis factor-α), translocation of nuclear factor-kappa B (NF-κB), and phenotypic transformations (phagocytic and migratory) in a dose-dependent manner. These inhibitory effects of PSE on microglia were supported by its regulatory effects on the CX_3_C chemokine receptor 1 (CX_3_CR1)/nuclear factor erythroid-2-related factor 2 (Nrf2) pathway. In particular, intragastric administration of PSE (100 mg/kg) considerably improved sickness, anxiety, and depressive-like behaviors in mice subjected to chronic restraint stress (CRS). Our results suggest that PSE has strong antineuroinflammatory and antidepressant properties, and the underlying mechanisms may involve not only the regulation of NF-κB translocation but also the normalization of the CX_3_CR1/Nrf2 pathway.

## Introduction

Neuroinflammation is a defense mechanism that allows an organism to adapt to harmful conditions, such as psychological stress and pathological infection in the brain. Excessive neuroinflammatory responses disrupt synaptic plasticity and neurotransmission, and such responses are commonly observed in patients with neurodegenerative and neuropsychiatric disorders. In particular, a number of studies have revealed a strong relationship between a high mental illness score and increased expression of translocator protein (TSPO, a marker of microglial-derived neuroinflammation) in patients with depressive disorders (Richards et al., 2018; Schubert et al., 2020).

Activated microglia produce a large amount of proinflammatory cytokines and chemokines via activation of the transcription factor NF-κB. These microglial-derived byproducts lead to demyelination, synaptic remodeling, and neuronal death. Notable findings showed that social stress increases the DNA binding affinity of NF-κB in patients with depressive disorders (Pace et al., 2006). Furthermore, overactivated microglial have been proposed to engulf neuronal synapses and dendritic spines, and the associations of overactivated microglia with depressive symptoms were recently investigated (Cao et al., 2021). As a unique neuron-glia crosstalk axis, the fractalkine-CX_3_CR1 pathway is an emerging target for the treatment of depressive disorder (Liu et al., 2020). Both clinical and preclinical studies reported that normalization of the fractalkine/CX_3_CR1 axis alleviates microglial activation and depressive-like symptoms (Merendino et al., 2004; Shangguan et al., 2020; Snijders et al., 2021). CX_3_CR1-deficient mice were more sensitive to LPS-induced inflammation and microglial-derived depressive-like behaviors (Corona et al., 2010; Wynne et al., 2010; Mattison et al., 2013; Hellwig et al., 2016). Therefore, regulating microglia is an important therapeutic strategy for depressive disorders.

*Pinus spp.* succinum has been prescribed for the treatment of amnesia, seizure, and anxiety symptoms in TCM. Volatile organic compounds identified in succinum, such as platambin and cycloseychellene, have been reported to inhibit inflammatory, chemotactic and phagocytic activity of innate immune cells (Silva-Filho et al., 2016; Zhang et al., 2017). These pharmacological activities were also observed in macrophages treated with succinic acid, which was isolated from succinum (Nissen et al., 2019). Exogenous treatment with succinic acid ameliorated neurodegeneration in the cerebellum of a mouse model of ataxia (Ferro et al., 2017), and its derivatives have antidepressant effects (Volchegorskii et al., 2019).

Herein, we investigated the anti-neuroinflammatory effects of succinum in BV2 microglial cells exposed to LPS and its antidepressant-like activity in mice subjected to CRS. We further explored the underlying mechanisms involving the NF-κB and CX_3_CR1 pathways.

## Materials and methods

### Materials and reagents

The following reagents were obtained from manufacturers: 4′,6-diamidino-2-phenylindole dihydrochloride (DAPI), ethyl alcohol, lipopolysaccharide (LPS; O111:B4), N-acetyl-L-cysteine (NAC), N-(1-naphthyl)-ethylenediamine dihydrochloride, phosphoric acid, sodium hydroxide, sulfanilamide, tetraethyl ethylenediamine (TEMED), Trizma base, and Tween 20 (Sigma‒Aldrich, St. Louis, MO, United States); antibiotic antimycotic solution, Dulbecco’s modified Eagle’s medium (DMEM), Dulbecco’s phosphate-buffered saline (DPBS), fetal bovine serum (FBS), and trypsin–ethylenediaminetetraacetic acid (EDTA) (Welgene, Daegu, Korea); 10% ammonium persulfate solution, radioimmunoprecipitation assay buffer (RIPA) buffer, and skim milk (LPS Solution, Daejeon, Korea); bovine serum albumin (BSA) (GenDEPOT, Barker, TX, United States); 4% paraformaldehyde (PFA), 10X Tris glycine buffer, and 10X Tris glycine-SDS buffer (XOGENE, Daejeon, Korea); protease inhibitor, phosphatase inhibitor, and RNA Later (Thermo Fisher Scientific, Waltham, MA, United States); sodium chloride and hydrochloric acid (Samchun, Seoul, Korea); methylene alcohol (Daejung Chemicals & Metals Co., Siheung, Korea); n-butanol (J.T. Baker, Mexico City, Mexico); Triton X-100 (Junsei Chemical Co., Ltd., Tokyo, Japan); normal chicken serum blocking solution (Vector Laboratories, Newark, CA, United States); and polyvinylidene fluoride (PVDF) membranes (Pall Co., Port Washington, NY, United States).

### Preparation of *Pinus spp.* extract

*Pinus spp.* was purchased from an herbal pharmaceutical company (Jeong-Seong Drugstore, Daejeon, Korea). *Pinus spp.* extracts were prepared as follows. Twenty grams of herbal powder was mixed with 200 ml of 30% ethyl alcohol and incubated with shaking for 72 h at room temperature. Then, the supernatants were filtered using Whatman filter paper (Advantec®, Tokyo, Japan). The filtrates were concentrated by rotavapor and then lyophilized. The final yield of PSE was 3.19% (w/w). The other nine candidates were also extracted using the same method. In addition, to compare the effectiveness with fraction, three fractions (BuOH; butanol, EtOAc; ethyl acetate and Hx; hexane) of *Pinus spp.* succinum were provided from National Institute for Korean Medicine Development (NIKOM, Gyeongsan, Korea).

### Fingerprinting analysis of PSE

To identify the chemical composition of PSE, we conducted gas chromatography (GC) and high-performance liquid chromatography (HPLC) analysis. The PSE was analyzed by GC‒MS with an Agilent 8890 N/5977B system (Agilent, Santa Clara, CA, United States). One microliter of PSE was injected at a temperature of 280°C with split ratios of 1/20 on an HP-5ms column (30 m × 0.25 mm, 0.25 μm: Agilent, Santa Clara, CA, United States). The temperature program was as follows: initial temperature 40°C (1 min), ramped at 10 °C/min to 300°C, and held for 5 min for a total run time of 30 min. Ultrahigh purity helium was used as a carrier gas at a 1.0 ml/min flow rate.

For the HPLC analysis, 10 mg of PSE was dissolved in 1 ml of distilled water, and the solutions were filtered (0.22 µm). Then, the filtrate was subjected to LC‒MS/MS using a Shimadzu LC-UV system (Shimadzu Co., Kyoto, Japan) equipped with an electrospray ionization source. Separation was performed on an Accela HPLC system using a Hector ODS column (250 × 4.6 mm, 5 μm; Rstech, Daejeon, Korea). The mobile phase conditions were prepared with 10 mM KH2PO4 in distilled water. The column was eluted at a flow rate of 0.8 ml/min with 100% A (isocratic). The photodiode array detector was set to measure a range of 208 nm.

### Cell culture and cytotoxicity assay

Murine microglial cells (BV2) and hippocampal neuronal cells (HT22) were cultured in DMEM supplemented with 10% FBS and 1% antibiotic-antimycotic solution (Welgene, Daegu, Korea). Both cell lines were incubated at 37°C in 5% CO_2_. BV2 (2 × 10^4^ cells/well) and HT22 (4 × 10^3^ cells/well) cells were seeded into a 96-well microplate for 12 h, and PSE or its fraction (PSF) was added and incubated for 24 h. The cytotoxicity was evaluated using a WST-8 assay kit (EZ-Cytox, DoGenBio, Seoul, Korea). The absorbance was measured at 450 nm using a UV spectrophotometer (Molecular Devices, Sunnyvale, CA, United States).

### Nitric oxide and proinflammatory cytokine assay

To evaluate the antineuroinflammatory effects of PSE or PSF, BV2 cells (2 × 10^4^ cells/well) were seeded and pretreated with different doses of PSE (10, 20, or 40 μg/ml), three PSF (10 μg/ml) or NAC (20 mM). After incubation for 2 h, the cells were exposed to 1 μg/ml LPS for 24 h. The supernatants were mixed with an equal volume of Griess reagent (1% sulfanilamide/0.1% N-(1-naphthyl)-ethylenediamine dihydrochloride/2.5% H_3_PO_4_). After incubation for 15 min at 37°C, the absorbance was measured at 540 nm using a UV spectrophotometer (Molecular Devices).

The cells were cultured under the same conditions as for the nitric oxide (NO) assay, and the levels of the proinflammatory cytokine tumor necrosis factor-alpha (TNF-α) in the supernatants were measured using a commercially available enzyme-linked immunosorbent assay (ELISA) kit (BD Biosciences, San Jose, CA, United States). The absorbance was read at 450 nm using a UV spectrophotometer (Molecular Devices).

### Phagocytosis assay

To evaluate the antiphagocytic effects of PSE, we assessed phagocytic activity using a phagocytosis assay kit (Cayman Chemical, Ann Arbor, MI, United States). BV2 cells were seeded at 2 × 10^5^ cells/well in 6-well plates and incubated for 12 h. The cells were pretreated with PSE or NAC for 2 h before exposure to 1 μg/ml LPS for 24 h; then, latex beads with rabbit IgG-fluorescein 5-isothiocyanate (FITC) conjugates (1:200) were added and incubated for 30 min, followed by a 1-min incubation with 4% PFA to fix the fluorescence of the phagocytosed beads. Fluorescence images were captured using an Axiophot microscope (Carl Zeiss, Jena, Germany).

### Cell migration assay

To investigate the antimigratory effects of PSE, BV2 cell cultures were wounded using a sterile 20 μL pipette tip to assess cell migration ability. The cells were cultured under the same conditions as for the NO assay, and the cell migratory activity over 24 h was determined by measuring the relative changes in the width of the wounds at the 0 h and 24 h time points using an Axiophot microscope (Carl Zeiss, Jena, Germany). The degree of cell migration is expressed as the percentage of that of the vehicle-treated cells.

### Immunofluorescence staining analysis

BV2 cells were cultured under the same conditions as for the NO assay, and then, the cells were sequentially washed with DPBS and fixed with 4% PFA for 20 min. In addition, the cells were permeabilized with 0.3% Triton X-100 for 10 min and blocked with 1% normal chicken serum in DPBS for 30 min. The cells were incubated with a CX_3_CR1 antibody (1:200, sc-377227; Santa Cruz, Dallas, TX, United States) at room temperature overnight. Then, the cells were incubated with goat anti-mouse IgG H&L (1:400, Alexa Fluor 488-conjugated, ab150077) for 2 h in the dark. The cells were subsequently exposed to DAPI (1:1000, D9542, Sigma) to stain the cell nuclei.

The day after the last day of CRS, the mice were transcardially perfused with 0.05% heparin (10 units/mL in PBS) followed by 4% PFA (pH 6.9). The removed brains were gradually cryoprotected in 10, 20 and 30% sucrose for 24 h each and were subsequently embedded in optimal cutting temperature compound (Leica Microsystems, Bensheim, Germany) in liquid nitrogen. They were cut into frozen coronal sections (35 μm) using a cryostat (CM3050_S, Leica), and sections were stored in free-floating buffer. After incubating with blocking buffer (5% normal chicken serum in PBS and 0.3% Triton X-100 for 1 h at 4°C), sections were adapted with anti-rabbit Iba-1 polyclonal (1:400, 019-19741, Wako Biologicals) primary antibodies overnight at 4°C. After washing with ice-cold PBS, the sections were incubated with a goat anti-rabbit (1:400; Alexa Fluor 488, ab150077) secondary antibodies for 2 h at 4°C. The sections were subsequently exposed to DAPI to stain the cell nuclei.

Immunofluorescence images were captured under an Axio-phot microscope (Carl Zeiss, Jena, Germany), and the signals were quantified using ImageJ 1.46 software (NIH, Bethesda, MD, United States).

### Western blotting analysis

To analyze the protein expression levels, BV2 cells were seeded at 3 × 10^5^ cells/well in 60 mm dishes and incubated for 12 h. After pretreatment with PSE or NAC for 2 h, the cells were exposed to 1 μg/ml LPS for 24 h. Total protein was extracted using Pro-Prep^TM^ protein extraction solution (iNtRON Biotechnology, Seongnam, Korea). The cytosolic and nucleic extracts of the cells were separated using NE-PER Nuclear and Cytoplasmic Extraction Reagents (Thermo Fisher Scientific, Carlsbad, CA, United States) according to the manufacturer’s instructions. The protein concentrations were determined using a bicinchoninic acid protein assay kit (Sigma‒Aldrich, St. Louis, MO, United States).

The cell lysates were separated by polyacrylamide gel electrophoresis and transferred to PVDF membranes. After the membranes were blocked in 5% skim milk in Tris-buffered saline (TBST; 0.05% Tween 20 in TBS) for 1 h, they were probed with primary antibodies against iNOS (1:1000, PA1-036, Thermo Fisher Scientific), COX2 (1:1000, 12282s, Cell Signaling, Danvers, MA, United States), CX3CR1 (1:1000, ab8021, Abcam, Cambridge, United Kingdom), p-Akt (1:1000, 9271S, Cell Signaling), Akt (1:1000, 9272S, Cell Signaling), p-ERK1/2 (1:1000, 9101S, Cell Signaling), ERK1/2 (1:1000, 9102S, Cell Signaling), Nrf-2 (1:1000, 12721, Cell Signaling), p-Nrf-2 (1:1000, ab76026, Abcam), HO-1 (1:1000, 374090, Merck, Darmstadt, DE), NF-κB p65 (1:1000, ab16502, Abcam), Lamin B1 (1:1000, sc-374015, Santa Cruz), and α-tubulin (1:1000, ab7291, Abcam) overnight at 4°C. The membranes were washed and incubated with HRP-conjugated anti-mouse (1:5000, to detect CX_3_CR1, Lamin B1, and α-tubulin) or anti-rabbit (1:5000, to detect iNOS, COX2, p-Akt, Akt, p-ERK1/2, ERK1/2, Nrf-2, p-Nrf-2, and NF-κB p65) antibodies for 45 min. The protein bands were visualized using an enhanced chemiluminescence (ECL) kit advanced. Protein expression was observed using the FUSION Solo System (Vilber Lourmat, Collegien, France). The band intensities were semiquantified using ImageJ 1.46 software (NIH, Bethesda, MD, United States).

### Animals and experimental design

Male C57BL/6J mice (8 weeks old, 21–23 g, n = 36) were purchased from Dae Han Bio Link (Co., Ltd, Eumseong, Korea). The mice had free access to water and food pellets (Cargill Agri Purina, Seongnam, Korea) and were housed in a room maintained at 23 ± 1°C on a 12 h:12 h light-dark cycle. The animal care and experimental protocols were approved by the Institutional Animal Care and Use Committee of Daejeon University (DJUARB 2021-021) and conducted following the Guide for the Care and Use of Laboratory Animals published by the National Institutes of Health (NIH).

After acclimation for 7 days, the mice were randomly divided into four groups (Total of nine mice per group; six mice for behavior test and three mice for molecular analysis, respectively): normal (no stress and distilled water), CRS (stress and distilled water), PSE (stress and 100 mg/kg PSE), and positive control (stress and 100 mg/kg NAC). PSE and NAC were dissolved in distilled water. One hour after oral administration of distilled water, PSE, or NAC, the mice were subjected to restraint stress by placement inside a 50 ml conical tube without access to food or water for 3 h daily for 21 days. The procedure was performed between 11:00 and 14:00 each day, and a 0.5 cm air hole was made to allow the animals to breathe. The CRS model was established as described previously (Buynitsky and Mostofsky, 2009).

### Nest building test

Sickness-like behavior was investigated with the nesting building test (NBT), as previously described (Deacon, 2006). Briefly, a total of 24 g of pressed cotton squares (ten squares per cage, 7 × 5 cm) were placed in the center of the floor of a cage house. The degrees to which the mice bit the squares, moved them into the corners, and nested with the squares overnight were scored from 0 to 5. The nest score of less than 2 was considered as a sickness condition, as described previously (Gaskill et al., 2013).

### Open field test

Anxiety-like behavior was evaluated using an open field test (OFT) as previously described (Ieraci and Herrera, 2006). The plastic box was contained in a square (40 × 40 × 30 cm), and the center of the field was defined by recording software. Each mouse was placed in the center of the area. We determined that mice exhibited anxiety-like behavior when they spent less than 50 s of their total time in the center zone. Their latency time in a central zone was recorded for 8 min with 50 lux lighting using a video camera connected to the corresponding smart 3.0 software (Panlab, Barcelona, Spain).

### Forced swimming test

Depressive-like behavior was examined using a forced swimming test (FST). The FST was performed using a cylindrical container 20 cm in diameter by 30 cm in height. The container was filled with water (24 ± 1°C) to a depth of 20 cm. The mice were individually placed in each container and allowed to swim for 6 min. Latency time was recorded when a mouse first assumed an immobile position, defined as a mouse floating in an upright position after 2 min, making only small movements to keep its head above water. The number of times a mouse assumed an immobile position was recorded for 4 min. We judged that the mice exhibited depressive-like behavior when the activity duration time was less than 60 s (Can et al., 2012).

### Statistical analysis

The data are presented as the means ± standard deviations. Statistical analysis was performed using GraphPad Prism 7 software (GraphPad, Inc., La Jolla, CA, United States). Statistical significance was determined using one-way variance analysis (ANOVA) followed by Dunnett’s test. In all analyses, *p* < 0.05 was considered significant.

## Results

### Fingerprinting of PSE

**FIGURE 1:**
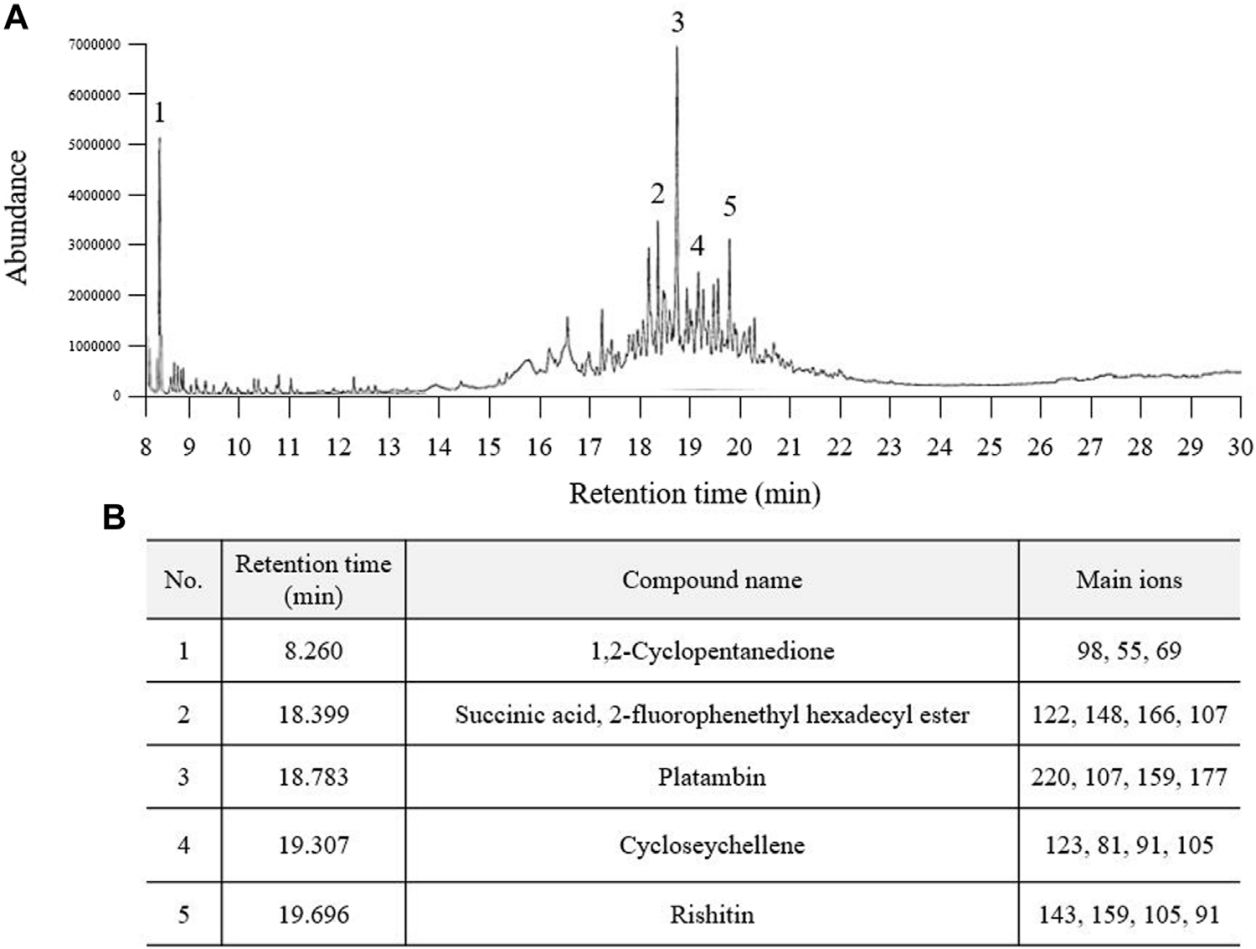
Fingerprinting analysis of Pinus spp. extract (PSE). PSE (30% ethanol extract) was applied to GC-MS and a chromatogram was obtained **(A)**. Five prominent peaks were detected at each retention time **(B)**.

In the GC‒MS analysis, a total of five major peaks were detected at retention times of 1,2-cyclopentanedione (8.260 min), succinic acid, 2-fluorophenethyl hexadecyl ester (18.399 min), platambin (18.783 min), cycloseychellene (19.307 min), and rishitin (19.696 min) at 280 nm (Figures 1A,B). Additionally, succinic acid (14.32 min) was detected using HPLC, and its content was 5.7 ± 0.16 mg/g PSE (Supplementary Figures S1A,B).

### Effects on cell viability, NO and NO-related molecules

**FIGURE 2:**
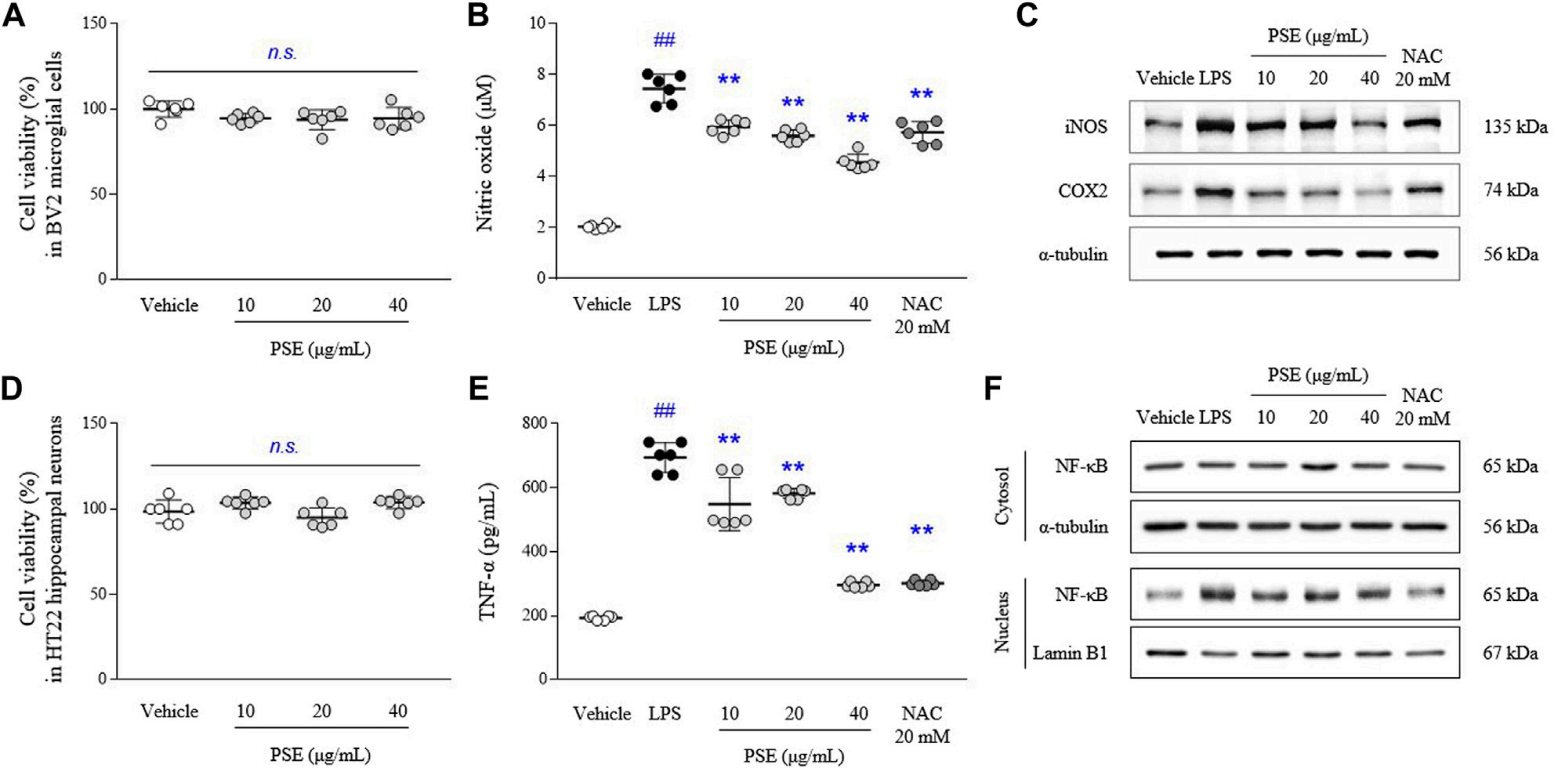
Cell cytotoxicity, NO, TNF-α and their-related molecules. BV2 and HT22 cells were treated with PSE for 24 h, or pretreated with PSE for 2 h before exposure to LPS (1 μg/ml) for 24 h (only BV2). The cytotoxicity of PSE was assessed using a WST-8 assay in BV2 **(A)** and HT22 **(D)** cells. The levels of NO **(B)** and TNF-α **(E)**, the protein expression of iNOS and COX2 **(C)**, and the cytosolic and nuclear levels of NF-κB **(F)** were measured. The data are expressed as the mean ± SD. ##*p* < 0.01 compared with the vehicle-treated cells; **p < 0.01 compared with the LPS-stimulated cells.

The PSE did not cause toxicity in BV2 and HT22 cells at concentrations up to 40 μg/ml (Figures 2A,D). LPS-induced increases in the levels of NO (3.6-fold, *p* < 0.05) were significantly inhibited by pretreatment with PSE (*p* < 0.05 for 10 μg/ml and *p* < 0.01 for 20 and 40 μg/ml; Figure 2B). PSE significantly decreased the protein expression levels of iNOS (*p* < 0.01 for all doses) and COX2 (*p* < 0.01 for all doses) that were increased by LPS induction (2.06- and 1.93-fold, *p* < 0.01 for both) compared with the vehicle alone (Figure 2C, Supplementary Figure S2C). NAC exerted similar effects as PSE. Regarding inhibitory effects against NO production, among three PSF, 10 μg/ml of EtOAc faction had a significant effect without cytotoxicity, but less than a high dose of PSE (Supplementary Figures S2A,B).

### Effects on proinflammatory cytokine and NF-κB nuclear translocation

The LPS-induced increases in the TNF-α levels (3.65-fold, *p* < 0.01) were significantly inhibited by pretreatment with PSE (*p* < 0.05 for 10 μg/ml and *p* < 0.01 for 20 and 40 μg/ml; Figure 2E).

LPS predominantly induced nuclear translocation of NF-κB, as shown by nuclear expression of NF-κB p65 (4.5-fold, *p* < 0.01), compared with the vehicle treatment, but the levels of NF-κB p65 expression in the cytosol were not changed. PSE pretreatment significantly inhibited the translocation of NF-κB p65 to the nucleus (*p* < 0.01 for all doses; Figure 2F, Supplementary Figure S2D). Subsequent pretreatment with NAC also exerted these similar effects.

### Effects on CX_3_CR1 and its-related molecules

**FIGURE 3:**
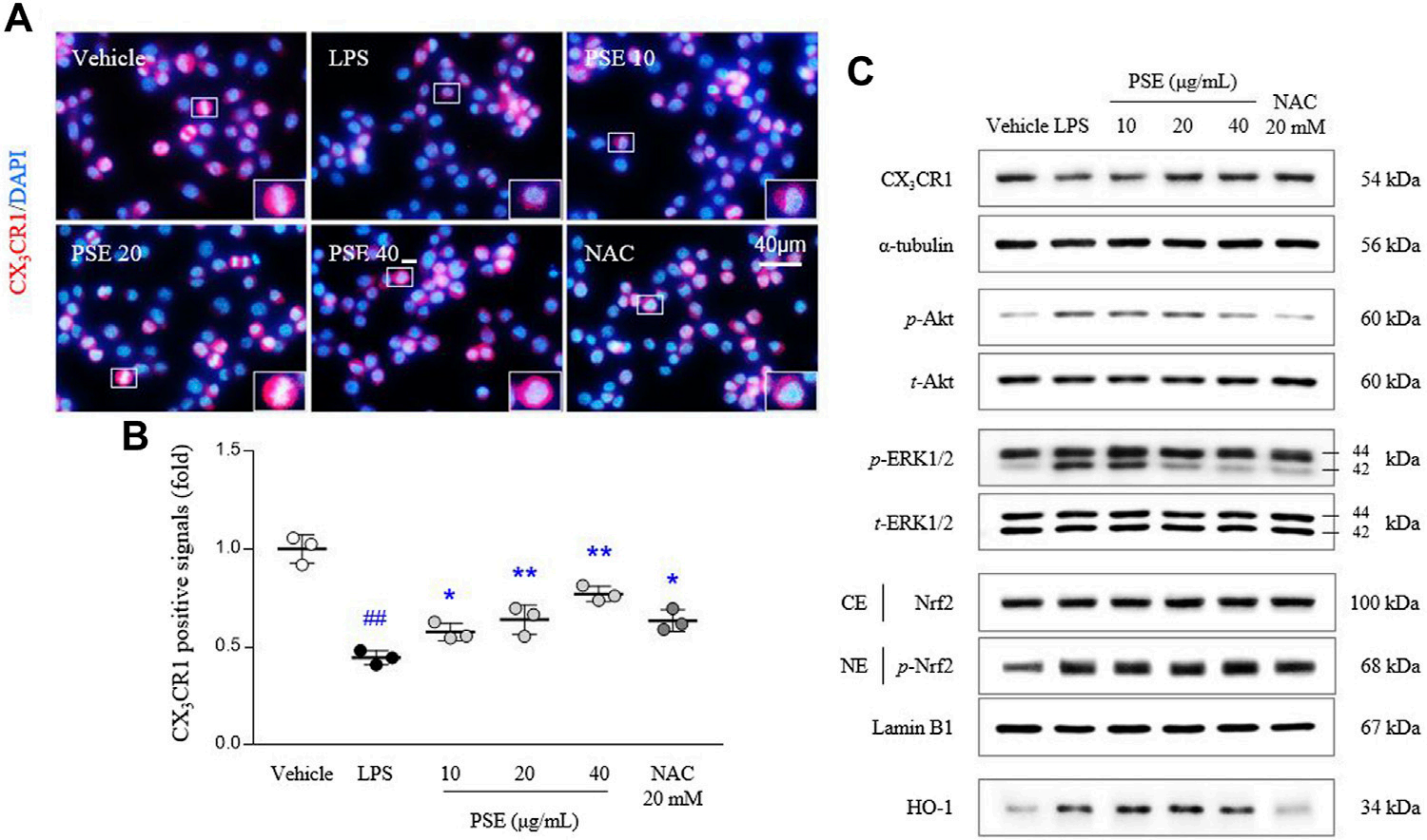
CX3CR1 signals and its related protein expressions. CX_3_CR1 fluorescence signals were semiquantified **(A,B)**, and protein expression levels of CX_3_CR1, p-Akt, Akt, p-ERK, ERK, the cytosolic and nuclear levels of Nrf2, and HO-1 **(C)** were determined. The data are expressed as the mean ± SD. ##*p* < 0.01 compared with the vehicle-treated cells; *p < 0.05 and **p < 0.01 compared with the LPS-stimulated cells.

The CX_3_CR1 level was inhibited by LPS treatment in BV2 cells, as evidenced by both the fluorescence (*p* < 0.01) and protein expression (*p* < 0.01) results. Pretreatment with PSE significantly reversed these decreases in the CX_3_CR1 levels (*p* < 0.01 for all doses and 20, 40 μg/ml, respectively) compared with LPS treatment (Figures 3A,B, Supplementary Figure S3B).

Signaling molecules downstream of CX_3_CR1, namely, the *p*-Akt/Akt ratio (3.92-fold, *p* < 0.01) and *p*-ERK/ERK ratio (1.71-fold, *p* < 0.01), were altered by treatment with LPS, and these changes in the *p*-Akt/Akt ratio (*p* < 0.05 for 10, 20 μg/ml and *p* < 0.01 for 40 μg/ml) and *p*-ERK/ERK ratio (*p* < 0.01 for all doses) were significantly normalized by PSE pretreatment (Figure 3C, Supplementary Figure S3C).

LPS significantly induced Nrf2 translocation to the nucleus, as shown by the nuclear expression of *p*-Nrf2 (1.17-fold, *p* < 0.01) compared with vehicle-treated cells. The nuclear translocation of *p*-Nrf2 was augmented by pretreatment with PSE (*p* < 0.05 for 40 μg/ml). In addition, PSE pretreatment increased the level of HO-1 protein expression (*p* < 0.05 for 10 and 20 μg/ml) more than LPS treatment (2.47-fold, *p* < 0.01; Figure 3C, Supplementary Figure S3C). NAC exerted similar effects as PSE.

### Effects on phagocytosis and cell migration

**FIGURE 4:**
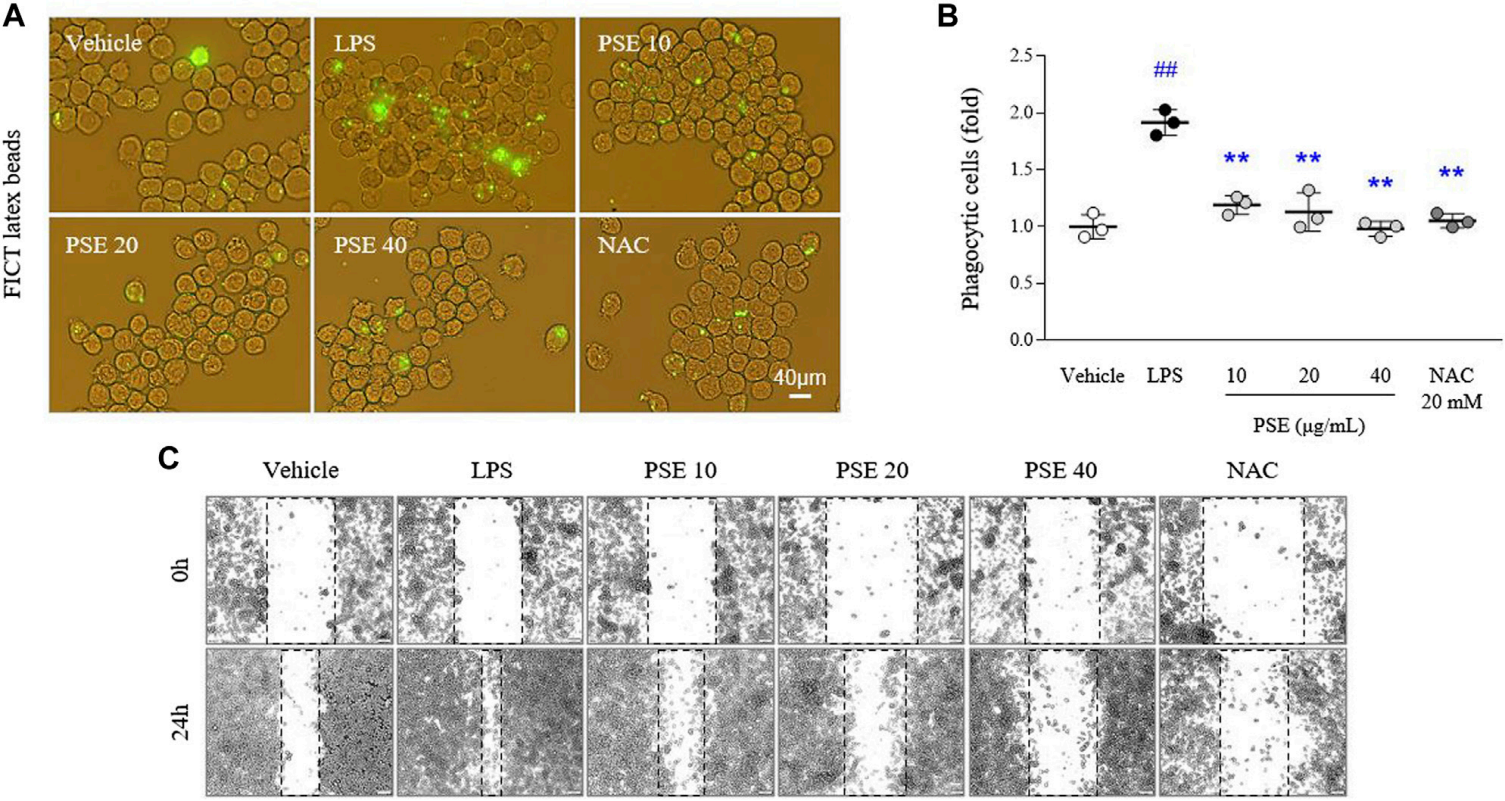
Phagocytic and migratory activities. Phagocytic activity was assessed using a phagocytosis fluorescent assay Kit (IgG FITC) **(A)**, and its intensities were semiquantifed **(B)**. Cells were scratched with a pipette tip, and then wound images were obtained at 0 h (i.e., immediately after scratching) and 24 h **(C)**. The data are expressed as the mean ± SD. ##*p* < 0.01 compared with the vehicle-treated cells; **p < 0.01 compared with the LPS-stimulated cells.

LPS increased intracellular FITC-positive signals (1.91-fold, *p* < 0.01) compared with vehicle treatment. The phagocytic activity increased by LPS was markedly decreased by pretreatment with PSE (*p* < 0.01 for all doses; Figures 4A,B). In addition, BV2 cell migration (1.7-fold, *p* < 0.01) was promoted by LPS treatment compared with the vehicle at the 24 h time point relative to the 0 h time point. The PSE pretreatment noticeably reversed this increased cell migration to a level similar to that of the vehicle-treated cells (*p* < 0.01 for all doses; Figure 4C, Supplementary Figure S3A). These effects were also observed following pretreatment with NAC.

### Effects on depression-like behaviors

**FIGURE 5:**
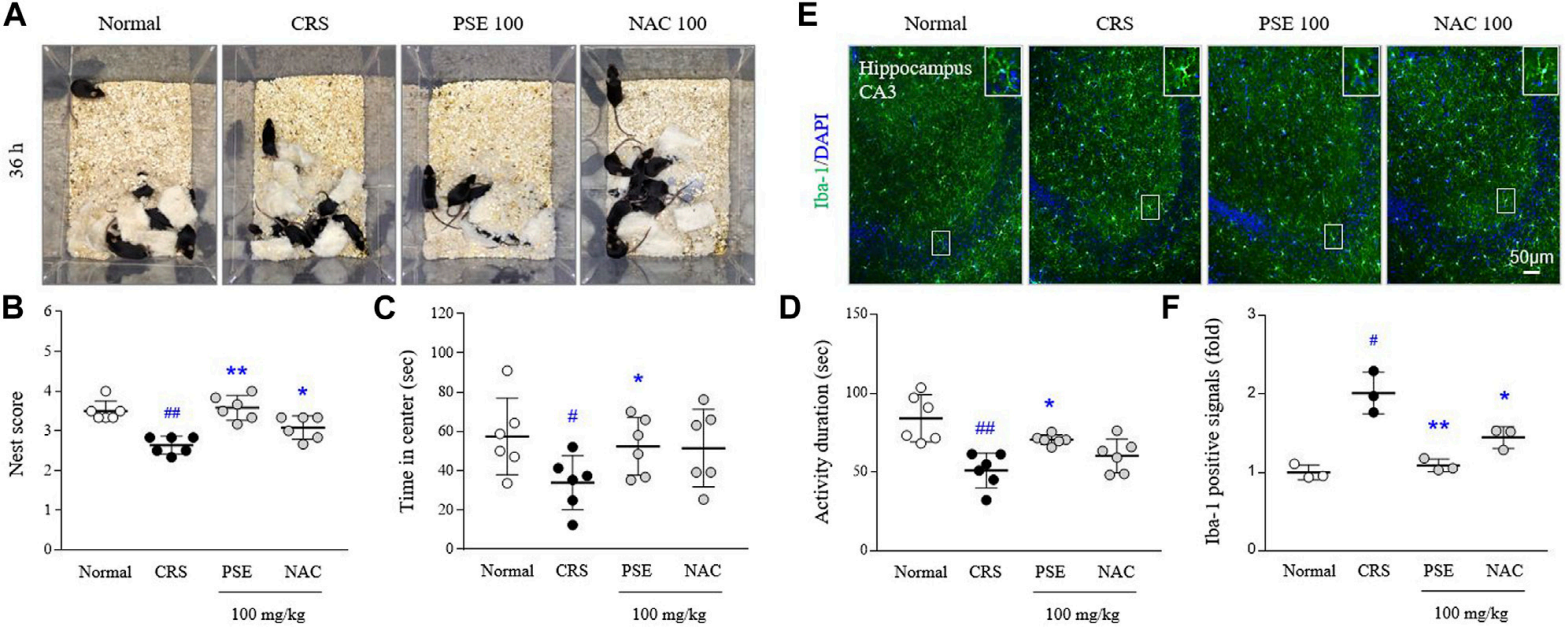
Sickness-, anxiety- and depressive-like behaviors, and microglial over-activity. Representative images of cotton nest construction **(A)** and nest score in the nest building test **(B)**, latency time in the center zone in the open field test **(C)**, and activity duration in the forced swimming test **(D)**. Immunofluorescence signals of Iba-1 in the hippocampus were measured **(E)**, and its intensities were semiquantifed **(F)**. The data are expressed as the mean ± SD. #*p* < 0.05 and ##*p* < 0.01 compared with the normal group; *p < 0.05 and **p < 0.01 compared with the CRS-induced group.

Exposure to CRS for 21 days exacerbated sickness-, anxiety-, and depressive-like behaviors compared with the normal group (*p* < 0.05 in all behavioral parameters). However, the administration of PSE significantly ameliorated CRS-induced depression behaviors, improving the nest scores in the NBT (*p* < 0.05), latency time in the center zone in the OFT (*p* < 0.05), and activity duration in the FST (*p* < 0.05; Figures 5A–D). NAC treatment only affected the score in the NBT.

### Effects on microglial over-activity in the hippocampus

CRS excessively activated hippocampal microglia, as evidenced by results from Iba-1 positive immunofluorescent signals (2-fold, *p* < 0.05) in hippocampal CA3 region. This over-activated microglia was considerably attenuated by administration of PSE compared with the CRS group (*p* < 0.05; Figures 5E,F), but not much in the NAC-treated group.

## Discussion

Neuroinflammation is considered a key target for the treatment of depressive disorder because existing antidepressants have critical limitations, such as poor responses and high remission and relapse rates (Andrews et al., 2012). Therefore, many groups are attempting to identify potential candidates with antineuroinflammatory and antidepressant properties.

To identify candidates that inhibit microglial-derived neuroinflammation, we screened ten herbal extracts using LPS-stimulated activated microglial cells. Candidates were prioritized based on TCM prescriptions used to treat mental illness (UNESCO, 2009) and the extracts of *Pinus spp.* succinum (PSE) exhibited superiority over the others in terms of its ability to inhibit microglial hyperactivation (data not shown). As expected, PSE pretreatment inhibited the LPS-stimulated increases in NO production and the expression of its enzymatic mediators, such as iNOS and COX2 (Figures 2B,C, Supplementary Figure S2C), in a dose-dependent manner, while PSE did not cause cytotoxicity in either BV2 microglial or HT22 hippocampal neuronal cells (Figures 2A,D). It is known that microglial-driven production of inflammatory cytokines, such as IL-1β and TNF-α, promotes pathogen neuroinvasion into the brain by damaging the blood‒brain barrier (BBB) or causing non-disruptive changes in the BBB (Osburg et al., 2002; Varatharaj and Galea, 2017). This antineuroinflammatory activity of PSE was also confirmed by the notable inhibition of NF-κB translocation to the nucleus and TNF-α production (Figures 2E,F, Supplementary Figure S2D). Microglial NF-κB activation contributes to the pathogenesis of Alzheimer’s disease via the accumulation of senile plaques (Wang C. et al., 2022) and major depressive disorder (Wang H. et al., 2022). Although NF-κB inhibitors are already considered agents with anti-neuroinflammatory properties, many scientists are concerned about problems associated with their application, such as nonselective disruption of NF-κB signaling (Dresselhaus and Meffert, 2019) and loss of pleiotropic roles in synaptic plasticity (O’mahony et al., 2006).

As an alternative to the above, recent studies have proposed the regulatory role of chemokine signaling pathways in microglial activity (Wolf et al., 2013; Trojan et al., 2017; Piirainen et al., 2021). The microglial-expressed chemokine receptor CX_3_CR1 is the sole receptor of fractalkine, which is secreted by neurons (Bazan et al., 1997; Ahn et al., 2019). Mice subjected to genetic ablation of CX_3_CR1 exhibit anxious behaviors and microglial abnormalities, including increased phagocytosis and aberrant synaptic pruning during brain development (Sahasrabuddhe and Ghosh, 2022). As expected, pretreatment with PSE considerably attenuated the reduction in CX_3_CR1 expression in microglia exposed to LPS (Figures 3A,B, Supplementary Figure S3B). Moreover, this pharmacological activity affected downstream signaling molecules (phosphorylation of Akt and ERK1/2) and the related anti-inflammatory Nrf2/HO-1 pathway (Figure 3C, Supplementary Figure S3C). One group indicated that CX_3_CL1, a fractalkine originating from hippocampal HT22 neuronal cells, activates the Akt and Nrf2/HO-1 pathways in BV2 microglial cells (Lastres-Becker et al., 2014). In contrast, enhancing microglial fractalkine/CX_3_CR1 signaling suppresses NF-κB translocation to the nucleus and proinflammatory cytokine production (Chidambaram et al., 2020). Clinical observations in moderate-severe or postmortem depressive patients have revealed an abnormal increase in serum fractalkine levels and CX_3_CR1 mRNA expression (Merendino et al., 2004; Snijders et al., 2021). Despite these seemingly opposite characteristics, substantial evidence suggests a pharmacological role of this pathway in microglial-mediated brain disorders (Pawelec et al., 2020).

The activated phenotype of microglia is known to trigger demyelination, dysregulation of neurotransmission, and synaptic pruning via phagocytic and migratory transformation (Kenny et al., 2006; Domercq et al., 2013; Lively and Schlichter, 2013; Zabel et al., 2016; Vilalta and Brown, 2018; Castro-Sánchez et al., 2019; Liu et al., 2021). As expected, pretreatment with PSE completely attenuated the LPS-induced effects on phagocytosis and migration (Figures 4A–C, Supplementary Figure S3A). In depressed and suicidal patients, the gene expression levels of Iba1 (marker of primed microglia) and MCP-1 (macrophage chemoattractant) were significantly increased in the dorsal anterior cingulate cortex (Torres-Platas et al., 2014). Based on our results from an *in vitro* study, we also investigated the antidepressant-like properties of PSE in a mouse model of depression established by CRS. As expected, PSE exerted pharmacological effects on sickness, anxiety, and depressive-like behaviors as well as inhibitory effect on microglial over-activation in hippocampal CA3 region (Figures 5A–F). Pooled data for two RCTs recently showed that minocycline, a specific microglial inhibitor, improved depressive symptoms and reduced the severity of anxiety in a total of 112 participants with major depressive disorders (Zazula et al., 2021). However, there is concern about other risks of using minocycline to treat anxiety and depression because it is also an antibiotic (Schmidt et al., 2021).

As an alternative and complementary medicine, *Pinus spp.* succinum has been prescribed for alleviating brain disorders and emotional abnormalities. The volatile organic compounds and terpenoids contained in this herb were shown to have anti-inflammatory properties (Silva-Filho et al., 2016; Sogo et al., 2021). In particular, it is known that succinic acid can penetrate the BBB, and exogenous treatment with succinic acid exerts ameliorative effects on mitochondrial dysfunction and neurodegenerative ataxia (Ferro et al., 2017). Moreover, succinic acid derivatives, such as emoxipine, reamberin and mexidol, exert acute antidepressant effects by inhibiting monoamine oxidase activity (Volchegorskii et al., 2019; Volchegorskii et al., 2020). For further process to identify the bioactive compound of succinum, we confirmed that EtOAc PSF was most similar to the PSE effect among the three fractions (Supplementary Figures S2A,B). In recent, isopimarane diterpenes showing anti-inflammation properties have been proposed as a bioactive compound of succinum, and it ameliorated cognitive impairment via inhibiting hippocampal apoptosis (Tungcharoen et al., 2020; Wei et al., 2022). As mentioned above, we speculate that terpenes-based compounds may involve with the anti-neuroinflammatory and antidepressant-like effects.

Here, we suggest that the ethanol extract of *Pinus spp.* succinum is a potential candidate for alleviating microglia-derived neuroinflammation and depression-like behaviors. Its underlying mechanism may involve regulating the NF-κB and CX_3_CR1 pathways. However, further investigations are required for the identification of corresponding compounds and confirmation of the specific underlying mechanisms.

## Data Availability

The original contributions presented in the study are included in the article/Supplementary Material, further inquiries can be directed to the corresponding authors.
