# Supplementary material for: Succinum extracts inhibit microglial-derived neuroinflammation and depressive-like behaviors
Source: Front Pharmacol. 2022 Aug 16;13:991243. doi: 10.3389/fphar.2022.991243 (PMC9425083; doi:10.3389/fphar.2022.991243)

Supplementary Material

## Supplementary Figure 1.

##
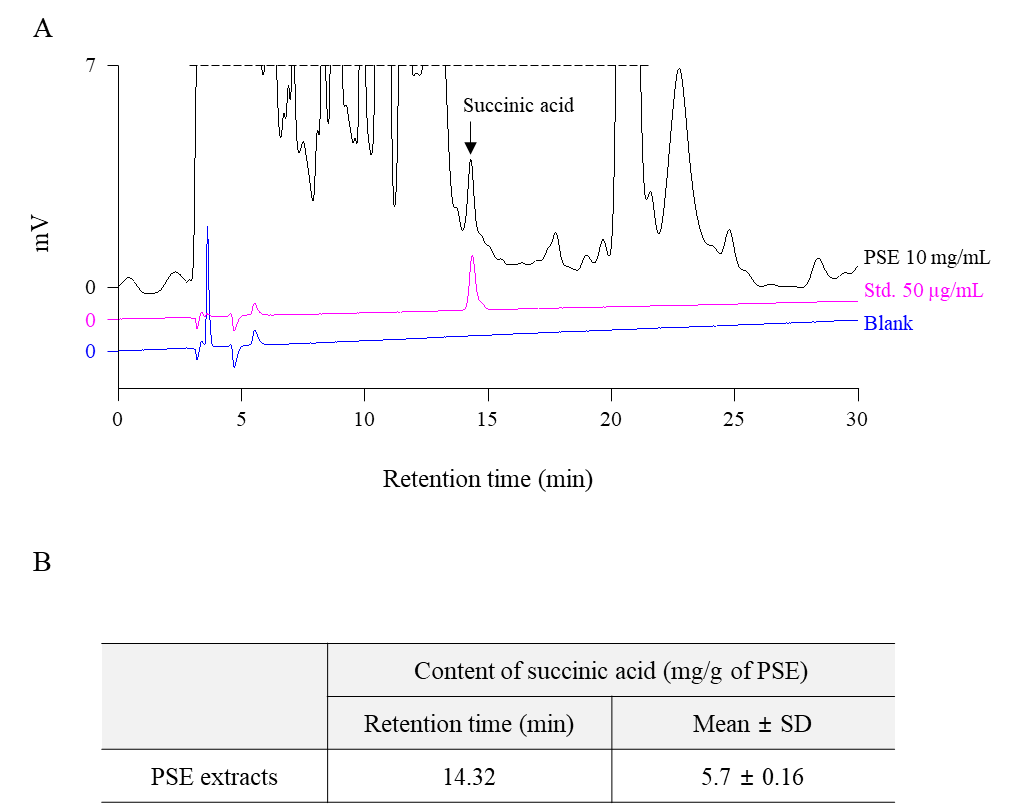


**Supplementary Figure 1.** **Fingerprinting analysis of the *Pinus spp.* succinum extracts (PSE).** Succinic acid was detected (A), and its retention time and area were semiquantified (B). PSE was subjected to HPLC‒MS, and a chromatogram was obtained at a wavelength of 208 nm.

## Supplementary Figure 2.

##
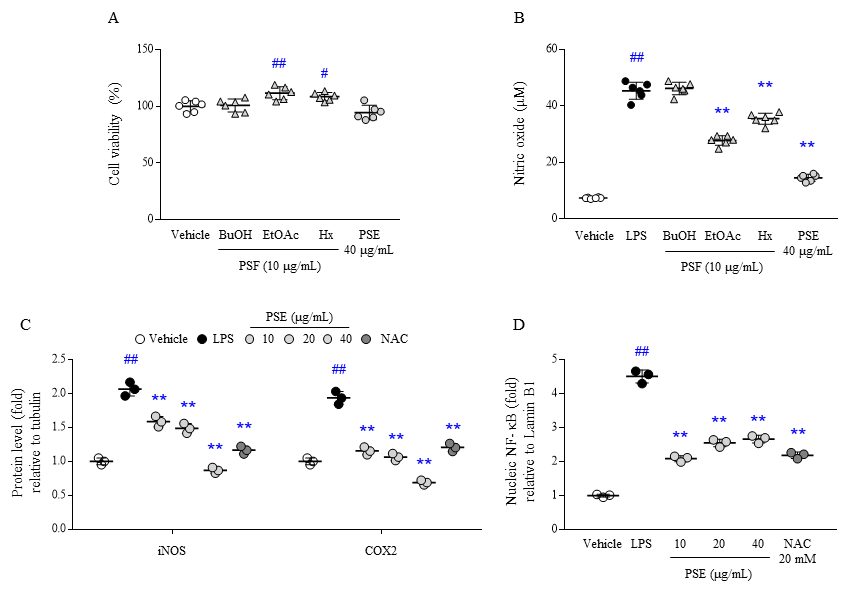


**Supplementary Figure 2.** **Cell cytotoxicity, NO, NO-related molecules, and nuclear NF-κB.** BV2 cells were treated with three PSF or PSE for 24 h and pretreated with three PSF or PSE for 2 h before exposure to LPS (1 µg/mL) for 24 h. The cytotoxicity of three PSF and PSE were assessed using a WST-8 assay (A). NO levels of three PSF and PSE (B) and protein expression relative to α-tubulin or lamin B1 against iNOS, COX2 (C), and nuclear NF-κB (D) were semiquantified. The data are expressed as the mean ± SD. # *p* < 0.05 and ## p < 0.01 compared with the vehicle-treated cells; ** p < 0.01 compared with the LPS-stimulated cells.

## Supplementary Figure 3.


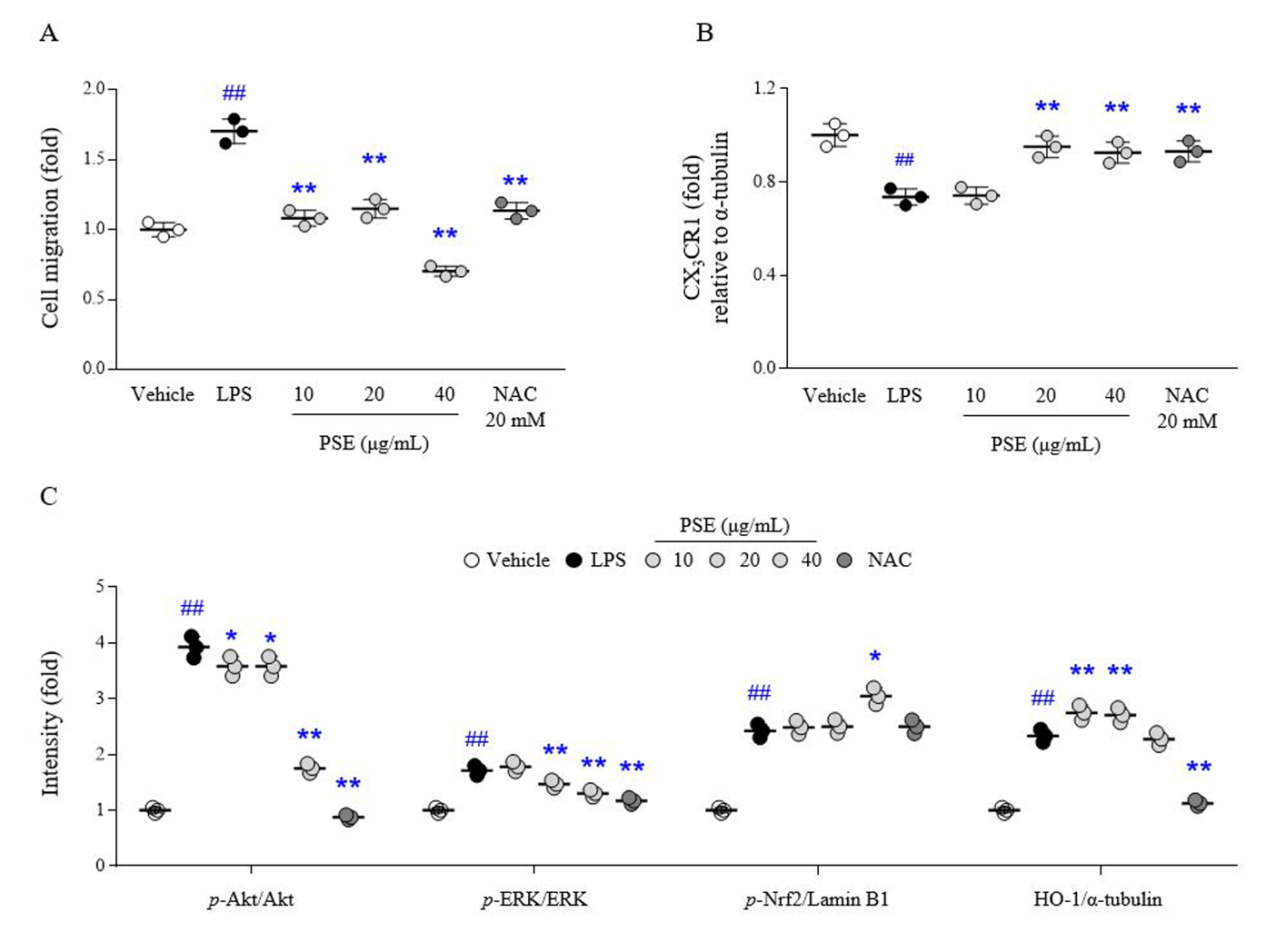


**Supplementary Figure 3. Cell migration, CX_3_CR1, and its related protein expressions.** Twenty-four hours after the scratch, the migration distance of BV2 cells was semiquantified (A). Levels of protein expression CX_3_CR1/α-tubulin ratio (B), p-Akt/Akt ratio, p-ERK/ERK ratio, p-Nrf2/Lamin B1 ratio, and HO-1/α-tubulin ratio (C) were semiquantified. The data are expressed as the mean ± SD. ## p < 0.01 compared with the vehicle-treated cells; * p < 0.05 and ** p < 0.01 compared with the LPS-stimulated cells.

Original blot data


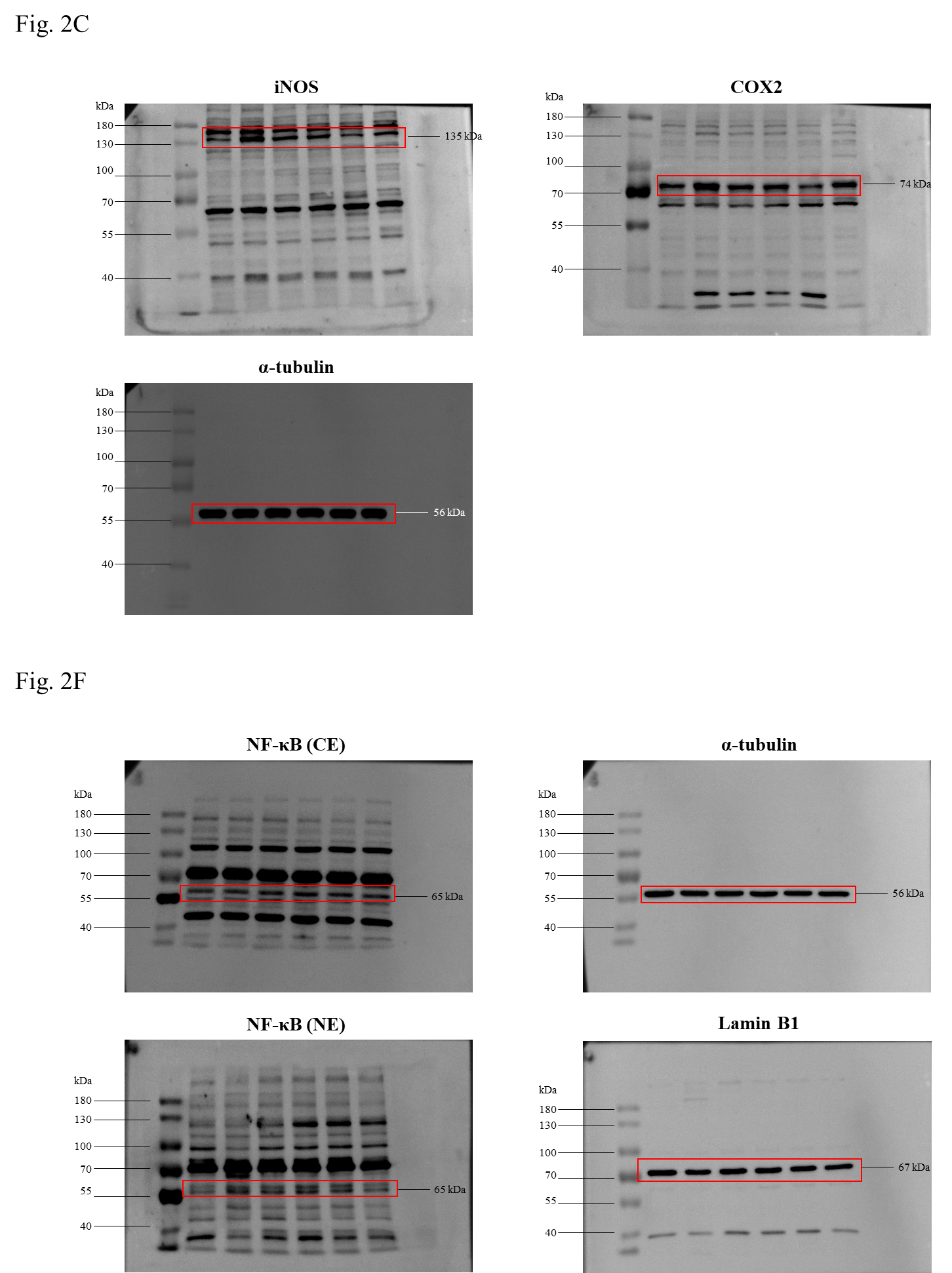


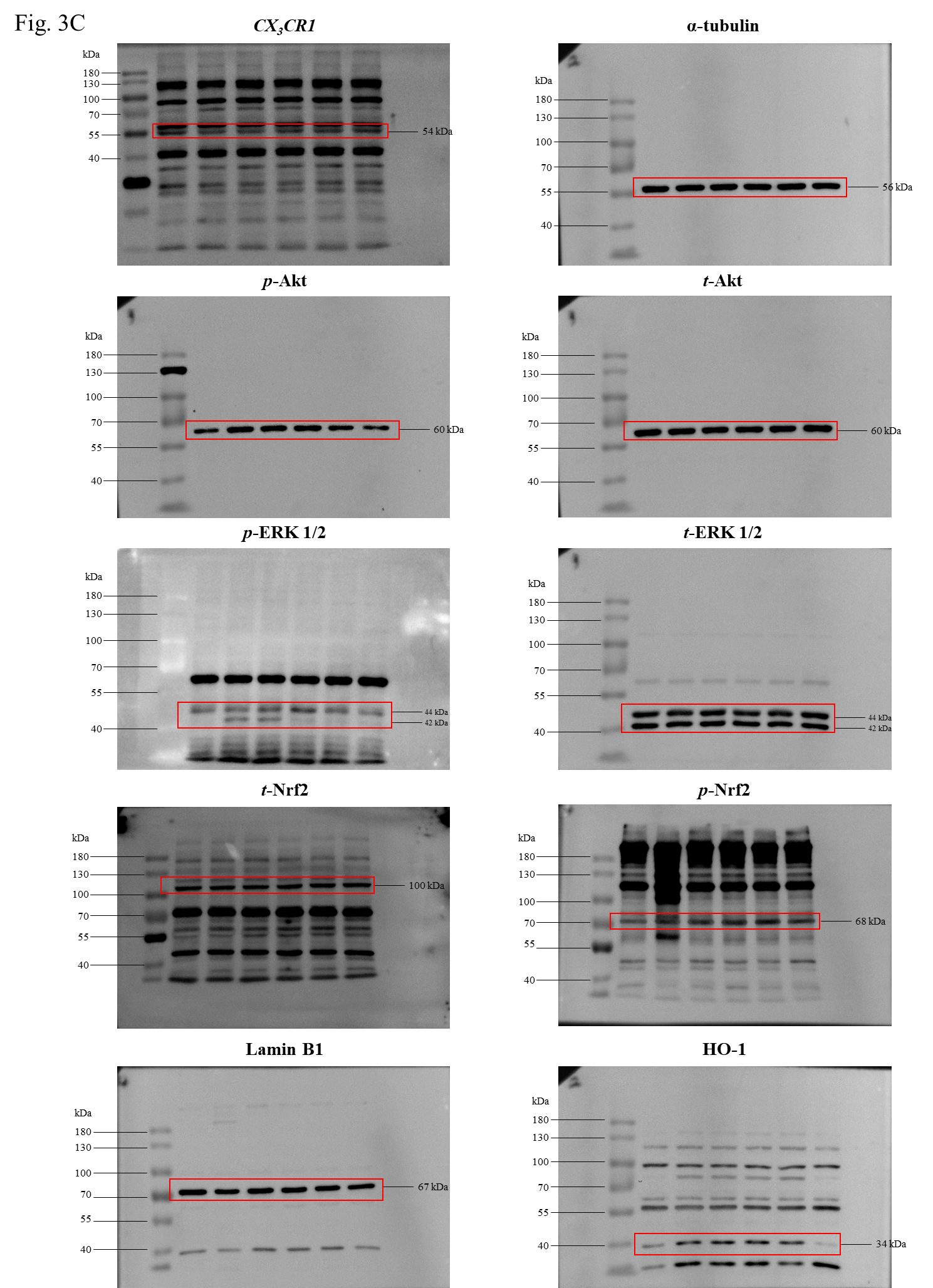

Supplement: Supplementary file 1 [file DataSheet1.docx]
